# Analysis of impact metrics for the Protein Data Bank

**DOI:** 10.1038/sdata.2018.212

**Published:** 2018-10-16

**Authors:** Christopher Markosian, Luigi Di Costanzo, Monica Sekharan, Chenghua Shao, Stephen K. Burley, Christine Zardecki

**Affiliations:** 1 Department of Molecular Biology and Biochemistry, School of Arts and Sciences, Rutgers, The State University of New Jersey, Piscataway, NJ USA; 2 RCSB Protein Data Bank, Institute for Quantitative Biomedicine, Rutgers, The State University of New Jersey, Piscataway, NJ USA; 3 RCSB Protein Data Bank, Skaggs School of Pharmacy and Pharmaceutical Sciences and San Diego Supercomputer Center, University of California, San Diego, La Jolla, CA USA; 4 Cancer Institute of New Jersey, Rutgers, The State University of New Jersey, New Brunswick, NJ USA

**Keywords:** Literature mining, Databases, Structural biology, Publishing

## Abstract

Since 1971, the Protein Data Bank (PDB) archive has served as the single, global repository for open access to atomic-level data for biological macromolecules. The archive currently holds >140,000 structures (>1 billion atoms). These structures are the molecules of life found in all organisms. Knowing the 3D structure of a biological macromolecule is essential for understanding the molecule’s function, providing insights in health and disease, food and energy production, and other topics of concern to prosperity and sustainability. PDB data are freely and publicly available, without restrictions on usage. Through bibliometric and usage studies, we sought to determine the impact of the PDB across disciplines and demographics. Our analysis shows that even though research areas such as molecular biology and biochemistry account for the most usage, other fields are increasingly using PDB resources. PDB usage is seen across 150 disciplines in applied sciences, humanities, and social sciences. Data are also re-used and integrated with >400 resources. Our study identifies trends in PDB usage and documents its utility across research disciplines.

## Introduction

The Protein Data Bank (PDB) is the single, global repository for structural data of the molecules of life. Understanding the 3D structure of a biological macromolecule is essential for understanding critical areas of science, including fundamental biology, medicine, energy, drug discovery, and education. As an established archive that continues to grow in size, the PDB provides an opportunity to study the impact of PDB data and resources in these different areas.

The PDB was established in 1971 to archive experimental data contributed by the new discipline of macromolecular crystallography, which was beginning to reveal three-dimensional (3D), atomic-level structures of biological macromolecules, including proteins, DNA, and RNA^1,2^. Today, the PDB also archives atomic coordinates and related experimental data from nuclear magnetic resonance spectroscopy and electron microscopy studies. Current archival holdings exceed 140,000 structures.

Since 2003, the Worldwide PDB (wwPDB) organization has managed the PDB archive and ensured that PDB data are freely and publicly available to *Data Consumers* around the globe^3,4^. Locally-funded, regional PDB Data Centers in the US^5^, Europe^6^, and Japan^7^ safeguard and disseminate PDB structures using a common data dictionary^8^ and a unified global system for data deposition-validation-biocuration^9^.

The Research Collaboratory for Structural Bioinformatics Protein Data Bank (RCSB PDB)^5,10^ has served as the US PDB Data Center since 1999. In 2017, RCSB PDB processed >6,200 new atomic level biomolecular structures plus experimental data and metadata contributed by PDB *Data Depositors* in the Americas and Oceania. wwPDB partners are together responsible for processing incoming data from elsewhere in the world. All data are available from the PDB archive; 11,124 new structures were released in 2017. The PDB archive and the RCSB PDB website (RCSB.org) are heavily used. During 2017, >680 million structure data files were downloaded from the archive by PDB *Data Consumers* worldwide. More than 1 million users from around the world benefited from open access to PDB data integrated with ~40 external resources at RCSB.org, providing rich structural views of fundamental biology, biomedicine, and energy sciences.

The most cited RCSB PDB publication, “The Protein Data Bank” by Berman *et al.*^5^, appeared in *Nucleic Acids Research* (*NAR)* in 2000. This inaugural article described the mission and vision of the resource and its operations, and provided data deposition and download instructions^11^. Berman *et al.* (2000) is routinely used to cite both the PDB data archive and the many services that the RCSB PDB provides to PDB *Data Depositors* submitting data (currently numbering >30,000) and PDB *Data Consumers* downloading data from the archive or using RCSB.org (currently >1 million). It has been heavily cited as noted by different reviews. A 2014 analysis^12^ ranked the inaugural article 92^nd^ among the top 100 most-cited research publications of all time and a 2017 study^13^ placed it 5^th^ among papers published since 2000.

By the end of 2016, Berman *et al.* (2000) had been cited by nearly 16,000 articles and other documents as recorded in the *Web of Science* database^14^. In 2017, RCSB PDB contracted with Clarivate Analytics to conduct an initial bibliometric analysis of citations to Berman *et al.* (2000) using in-house tools and proprietary data made available on a fee-for-service basis. Their report was made public by the RCSB PDB during the same year^13^.

The Clarivate analysis was *per force* limited to the activities of researchers who were referencing the *Nucleic Acids Research* publication in order to cite the RCSB PDB, specific PDB structures, and/or the PDB archive as a whole. Complicating matters further, many PDB *Data Consumers* do not cite Berman *et al.* (2000), electing instead to mention the RCSB PDB website (i.e., RCSB.org) or refer to individual PDB structures directly using a unique 4-character identifier (e.g., PDB ID 1vol)^15^. Some researchers have even grown so accustomed to using the RCSB PDB and the PDB archive that it is never mentioned in their publications, although close reading of their publications reveals reliance on the resource.

Herein, we report the results of systematic analyses of the types of published research citing the Berman *et al.* (2000) reference as well as PDB archival data reuse by >400 other online biodata resources. In addition, we relate these findings to outcomes from an online RCSB PDB user survey and the Clarivate study to provide a comprehensive picture of the breadth and depth of the impact on the scientific community of the RCSB PDB and the PDB archive.

## Results

### Exploration of Research Category

Between 2000 and 2016, the inaugural RCSB PDB publication, Berman *et al.* (2000), has garnered 15,711 citations in the Web of Science (Data set, Data Citation 1)^14^. Citing documents include articles, proceedings papers, reviews, book chapters, editorial material, and software reviews. The 2017 citation analysis^13^ by Clarivate Analytics focused on the >14,000 journal articles citing “The Protein Data Bank.” Web of Science assigns journals exclusively to 1 of 22 Essential Science Indicators (ESI) Subject Categories in science and social sciences. Citing articles were found in all ESI Subject Categories except Space Science; in particular, ESI categories Biology & Biochemistry, Chemistry, Molecular Biology & Genetics, and Computer Science contained the most citing articles. Clarivate determined that these citing articles were generally considered to be “high-quality” across fields. As citation rates vary between research areas and journal impact factors, Clarivate considers a variety of factors (e.g., world average citations per publication for the year, journal category, document type) to “normalize” citations in order to compare impact across disciplines. Using their Category-normalization, the citation impact of articles was at least the world-average except in the category of Psychiatry/Psychology. The greatest impact was seen in Computer Science, with a citation-based impact that was twice the world average.

In 2017, an online user survey was held to collect information about the research interests of RCSB PDB users (2017 RCSB.org Survey Data, Data Citation 1). 86% of respondents reported completing a Bachelor’s degree or higher. Subject area interests for all respondents mirrored the Web of Science analysis by Basner^13^. Most users predominantly identified their research interests as Biology & Biochemistry (72%), Molecular Biology & Genetics (31%), Chemistry (23%), Microbiology (8%), Computer Science (8%), and Immunology (6%). This finding strengthens the argument that the PDB is primarily used by individuals within the biological sciences and those working in fields with interdisciplinary applications. It is critical to note that 59% of survey respondents reported never having deposited a structure in the PDB, showing that the PDB plays a vital role in education and/or provides utility for researchers who are consumers of 3D structural information on biomolecules. In fact, 62% of all respondents report using the PDB-101 educational series *Molecule of the Month*.

**Figure 1:**
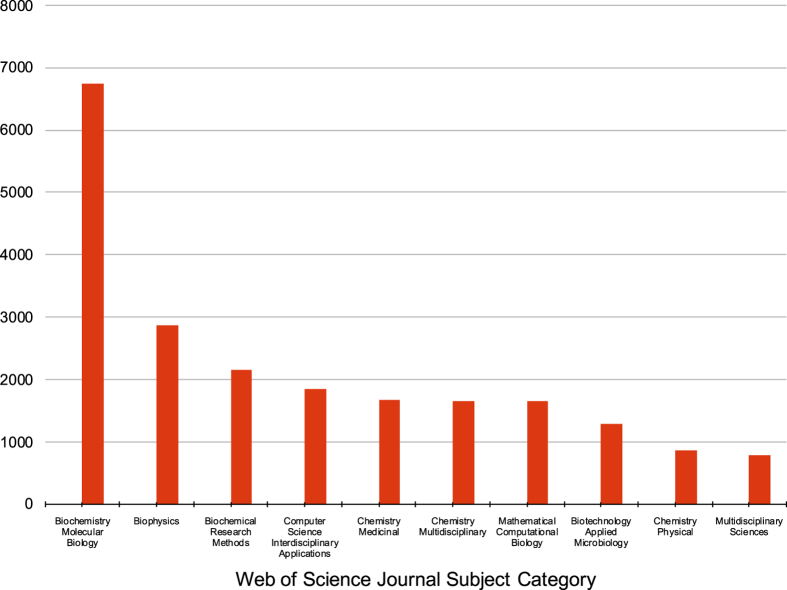
Number of publications for the top-assigned Web of Science Journal Subject Category for all documents (2000–2016) citing the inaugural Berman *et al.* (2000) reference. *Biochemistry Molecular Biology* is the largest category (6,735 publications), followed by *Biophysics* (2,872), *Biochemical Research Methods* (2,161), *Computer Science Interdisciplinary Applications* (1,852), *Chemistry Medicinal* (1,666), *Chemistry Multidisciplinary* (1,660), *Mathematical Computational Biology* (1,656), *Biotechnology Applied Microbiology* (1,297), *Chemistry Physical* (871), and *Multidisciplinary Sciences* (789).

Web of Science also utilizes more granular Journal Subject Categories. To obtain a more detailed understanding of PDB usage, we used the online Web of Science interface^14^ to determine that the citing articles were themselves published in journals across 154 Journal Subject Categories (out of a possible 252). The top ten Journal Subject Categories (Fig. 1) revealed the expected large number of assignments associated with experimental structure determination: *Biochemistry Molecular Biology* (22%), *Biophysics* (9%), *Biochemical Research Methods* (7%), and *Biotechnology Applied Microbiology* (4%). *Chemistry Medicinal* (5%) is almost certainly prominent because of the importance of structural data in drug discovery. *Chemistry Physical* (3%) is also most likely common because it utilizes structural data to interpret thermodynamics and kinetics of macromolecules. However, the presence of *Computer Science Interdisciplinary Applications* (6%) and *Mathematical Computational Biology* (5%) among the top 10 underscore the widespread use of PDB data in conjunction with computational techniques.

**Figure 2:**
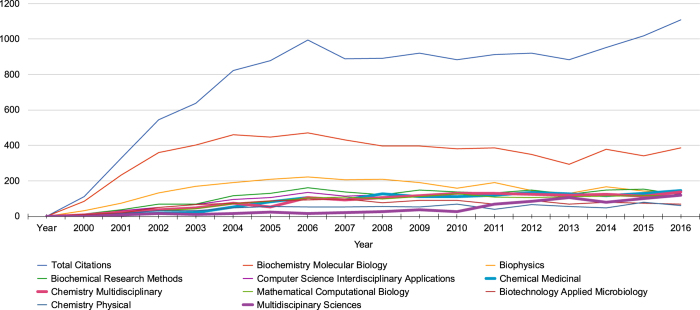
Number of articles citing the inaugural Berman *et al.* (2000) reference each year. Total number of articles is shown in blue; the top Journal Subject Categories are below. Growth in the areas of *Chemical Medicinal*, *Chemistry Multidisciplinary*, and *Multidisciplinary Sciences* is increasing (shown in bold); the number of articles in the areas of *Biochemistry Molecular Biology*, *Biophysics*, and *Biotechnology Applied Microbiology* do not have statistically significant growth.

Since 2004, the annual number of citations of Berman *et al.* (2000) is consistently high, with an average of ~940 articles per year and no evidence that overall usage is on the verge of decline. Fig. 2 charts the total number of citing articles per year, with the top Journal Categories mapped underneath. Throughout 2004–2016, the majority of articles fall within the *Biochemistry Molecular Biology* category, while that number appears to be decreasing slowly in favor of other category topics.

**Figure 3:**
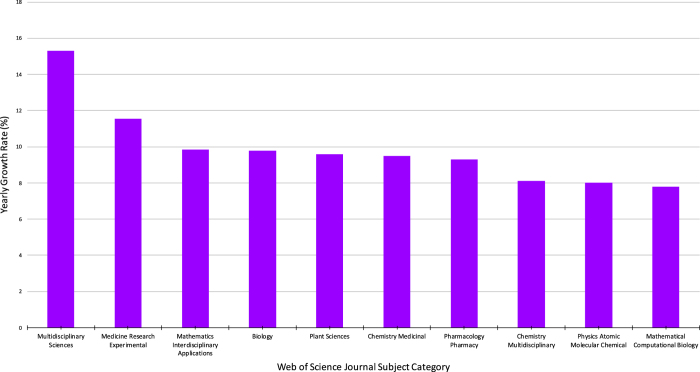
The top Web of Science Journal Subject Categories demonstrating the greatest yearly growth in all documents citing the Berman *et al.* (2000) reference (2000–2016). The study compares 34 categories with at least 100 citations. Growth rate was calculated as the slope coefficient of the linear regression model between the number of citations in the category and year of publication, starting with the first year an article appeared, and expressed as a normalized percentage of the average yearly publication of that category. Multidisciplinary Sciences has grown at the greatest rate (15.3%), followed by Medicine Research Experimental (11.6%), Mathematics Interdisciplinary Applications (9.8%), Biology (9.8%), Plant Sciences (9.6%), Chemistry Medicinal (9.5%), Pharmacology Pharmacy (9.3%), Chemistry Multidisciplinary (8.1%), Physics Atomic Molecular Chemical (8.0%), and Mathematical Computational Biology (7.8%).

Analyses of the yearly growth rate of each Journal Subject Category provides information relating to the fastest growing research areas throughout 2000–2016. This perspective is important in understanding the future direction of PDB usage. Overall, the categories with the most citations were among categories with the smallest yearly growth rates (*Biochemistry Molecular Bi*ology, 1.1% and *Biophysics*, 1.5%). Other categories, with fewer total citations, exhibit much greater yearly growth rates (*Multidisciplinary Sciences*, 15.3% and *Medicine Research Experimental*, 11.6%). This trend suggests that the way the PDB data are being used is changing. It is remarkable that the categories *Chemistry Medicinal, Chemistry Multidisciplinary,* and *Mathematical Computation Biology* appear in the top ten Journal Subject Category by both count (Fig. 1) and growth rate (Fig. 3).

Articles citing Berman *et al.* (2000) are published in journals categorized in >100 additional subject categories, indicating the broad range of PDB data utilization. Examples drawn from categories that are not directly related to structural biology demonstrate both the unique applications and the breadth of influence of the PDB archive. Industrial-focused categories include *Polymer Science, Agriculture Dairy Animal Science*, *Energy Fuels*, and *Forestry*. An example from *Agronomy* studied flavonoid antifungal activity against *Aspergillus ochraceus* through *in silico* analysis of small molecule interactions with kinase structures in the PDB^16^. In *Energy Fuels*, one study refers to modeling protein structures using PDB data to design more efficient biocatalysts for industrial applications^17^. Other Medical categories that reference PDB include *Oncology*, *Infectious Diseases*, *Nutrition Dietetics*, and *Veterinary Sciences*. An example from *Health Care Sciences Services* calls for the establishment of a central repository of raw data for the human brain project, citing the PDB as an example of “highly successful bioinformatics efforts”^18^. Within the tail of the distribution of research areas citing, we found publications in unexpected categories, such as *Optics*, *Telecommunications*, and *Social Issues*. For example, a *Management* study exhibits the importance of management science tools to address issues in bioinformatics and cites the PDB as an important tool in disseminating macromolecular structural data^19^. Interestingly, publications were also noted in entirely unexpected disciplines and fields, such as *Art*^20–24^, *Logic*^25–27^, and *Business Finance*^28^.

**Figure 4:**
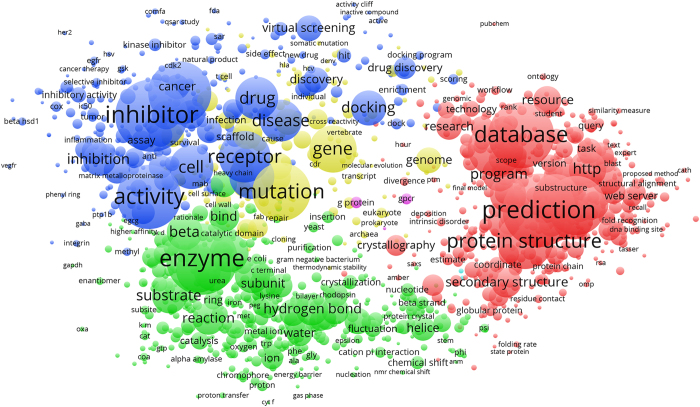
Network visualization of term occurrences extracted from abstracts and titles of 2000-2016 publications citing the inaugural Berman *et al.* (2000) reference. Figure created using VOSviewer^46^. A threshold cutoff of 30 as number of term co-occurrence was used. The location of citation keywords is based on their overall position within the network; keywords located in more common regions of the map have higher network connectivity, i.e., they are more interconnected with surrounding keywords. Darker colors and font size represent keywords that appear more frequently among citations. Keywords are clustered in four main regions: red corresponds to keywords representing “computational” use of the data; green corresponds to 3D-structure and mechanism of action; blue corresponds to function; and yellow corresponds to keywords related to genetics and genomics.

Creation of a co-occurrence network map reiterates the breadth of journal subject categories. The titles and abstracts of the ~15,000 articles published between 2000–2016 citing Berman *et al.* (2000) were used to generate a keyword map based on frequency of occurrence (Fig. 4; Network Visualization Map Data, Data Citation 1). Terms were concentrated in four main regions. On the right (red) are keywords linked to computational usage, including “prediction” (3,611 total occurrences), “database” (3,029), and “protein structure” (2,339). The bottom region (green) describes the 3D PDB structures themselves: “substrate” (1,127), “hydrogen bond” (978), “reaction” (952), and “subunit” (770). A dominating term in this region is “enzyme” (3,440), which falls between keywords linked to structure (green) and function (blue). Other terms in this region of the keyword plot include “inhibitor” (3,513), “activity” (2,901), “receptor” (2,046), and “drug” (1,536). The center of the map (yellow) reflects the impact of PDB data on overall understanding of genetics and genomics, with the terms “mutation” (2,504) and “gene” (1,780).

### PDB Usage in Other Databases

An important role of the PDB archive is to make structural data freely available for reuse by other resources^29^. The *Nucleic Acids Research* (*NAR*) Online Molecular Biology Database Collection^30^, a compilation of biological databases updated annually, was assessed to determine PDB usage by other resources. As of January 2018, the collection has 1,737 active databases organized into 15 NAR categories^31^. A review of this collection shows that 429 active bioinformatics resources utilize PDB data across 14 of the 15 categories (Table 1 (available online only); NAR Molecular Biology Database Collection Data, Data Citation 1). These databases encompass 44 subcategories, highlighting the variety of applications of PDB data. These databases add value to PDB data and function as resources for protein-protein interactions (e.g., AffinDB)^32^, enzymes (e.g., MultiTaskDB)^33^, metabolic pathways (e.g., MMCD)^34^, signaling pathways (e.g., REPAIRtoire)^35^, mapping cancer mutations in proteins (e.g., Cancer3D)^36^, antibody structure (e.g., SAbDab)^37^, viral capsid structures (e.g., VIPERdb)^38^, yeast genomics (e.g., SGD)^39^, drug-binding sites (e.g., sc-PDB)^40^, and more.

From the 1,737 resources in the Online Molecular Biology Database Collection, the leading 108 databases used most intensively across the scientific community have been identified by *NAR* as the “golden set” of successful resources^41^. Our review revealed that 52 of these elite databases incorporate PDB data, demonstrating the importance of the PDB archive in resources widely used in the biological research community.

## Discussion

While the PDB was initially established as an archive to serve structural biologists by hosting and providing access to their experimental data, bibliometric analyses of the heavily-cited Berman *et al.* (2000) reference^5^ demonstrate that the PDB has grown to serve a much more diverse community of users. While expected Web of Science journal categories, such as Biochemistry Molecular Biology and Biophysics, continue to generate the most citing publications per year (Fig. 1), disciplines such as Mathematical Computational Biology, Chemistry Medicinal, and Computer Science Interdisciplinary Applications are generating citations at much greater growth rates (Fig. 2). Moreover, keywords associated with structure determination and description were prevalent among citing publications in the early 2000s, but keywords pertinent to drug discovery, then genetics and genomics, and most recently protein structure prediction have come to the fore. Usage across disciplines is unmistakably confirmed by review of PDB usage within the *NAR* Online Molecular Biology Database Collection^41^.

Widespread usage of PDB data is also in line with survey results, which demonstrated that only a minority of RCSB PDB users have ever contributed a structure to the archive. The combination of these analyses indicates widespread use of the RCSB PDB and PDB archive in education and research, going well beyond studies of individual structures.

## Methods

Publication data for articles and documents from 2000–2016 that cited the Berman *et al.* (2000) reference^5^ were exported from the Web of Science (Data set, Data Citation 1)^14^. Each publication can be assigned to more than one category and/or more than one country. Exported data were analyzed using Microsoft Excel in Fall 2017.

The data examined are limited to those citing this inaugural reference, and future work could show either expanded usage or usage in greater detail. Going beyond the scope of this work, additional analyses could be performed to include publication data for articles citing the wwPDB^3^, wwPDB data centers PDBe^6^ and PDBj^7^, wwPDB partner BioMagResBank^42^, as well as the NAR articles describing the wwPDB partners that are published regularly (for examples see recent articles for RCSB PDB^10^, PDBe^43^, PDBj^7^). Other analyses of PDB usage have examined citations and patents^44^ and usage of URLs in the literature^15^. The data set under consideration was limited to Web of Science data for articles citing the inaugural RCSB PDB publication to be consistent with the Clarivate Analytics report^13^.

The User Survey was hosted at SurveyMonkey during the month of October 2017, and promoted on RCSB.org, PDB101.rcsb.org, message boards, and social media. A total of 681 responses were received from high school students and teachers, undergraduates, graduate students, postdoctoral scholars, and faculty/staff (2017 RCSB.org Survey Data, Data Citation 1).

A co-occurrence network map of keywords from the same set of publication data was created using the VOSviewer server (Version 1.6.5; www.vosviewer.com)^45,46^. Approximately 227,000 keywords were extracted from citation titles and abstracts (Network Visualization Map Data, Data Citation 1). A network was computed for a total of 2,460 terms selected by the full-counting method and relevance scoring as implemented within VOSviewer. For analysis, we reviewed co-occurrence network maps for thresholds between 5 and 40. A total of 60% total number of terms with a default cutoff of 30 as the number of term co-occurrence is shown.

The online descriptions and related publication abstracts for the resources listed in the *NAR* Online Molecular Biology Database Collection^42^ as of January 2018 were text mined for the words “protein data bank,” “PDB,” and other terms related to protein structure. Only the 1688 databases with available abstracts were mined. Results were manually reviewed to confirm usage of PDB data and website availability (NAR Molecular Biology Database Collection Data, Data Citation 1).

### Code and data availability

The following have been uploaded to FigShare: bibliographic data for articles citing the inaugural Berman *et al.* (2000) reference (with permission from Clarivate Analytics); anonymized user survey data; VOSviewer map file; and the current list of *NAR* Online Molecular Biology Database Collection resources that utilize PDB data.

The scripts used to obtain the initial data for the analysis of the *NAR* Online Molecular Biology Database Collection are available from GitHub: https://github.com/rcsb/review-NAR-Databases.

## Additional information

**How to cite this article**: Markosian, C. *et al.* Analysis of impact metrics for the Protein Data Bank. *Sci. Data*. 5:180212 doi: 10.1038/sdata.2018.212 (2018).

**Publisher’s note**: Springer Nature remains neutral with regard to jurisdictional claims in published maps and institutional affiliations.

## Figures and Tables

**Table 1:** Distribution of the 429 active resources in the *NAR* Molecular Biology Database Collection that utilize PDB archive data across major categories (bold) and subcategory (italics), and corresponding resources in the “golden set”^41^ of the *NAR* Molecular Biology Database Collection.

***NAR*** **Category and Subcategory**	**Number that Utilize PDB Data**	***NAR*** **Golden Set**
**Structure Databases**	**107**	
*Protein Structure*	81	MMDB^47^, PDBe,^6^ PDBsum^48^, RCSB PDB,^5,10^ SCOP2^49^, SUPERFAMILY^50^, SWISS-MODEL Repository^51^
*Small Molecules*	11	ChEBI^52^, PubChem^53^
*Nucleic Acid Structure*	8	
*Carbohydrates*	7	
**Protein Sequence Databases**	**95**	
*Databases of Individual Protein Families*	42	GPCRDB^54^, MEROPS^55^
*Protein Domain Databases; Protein Classification*	14	CDD^56^, CATH^57^, Pfam^58^, SMART^59^
*Protein Sequence Motifs and Active Sites*	15	ELM^60^, PROSITE^61^
*Protein Properties*	10	dbPTM^62^
*Protein Localization and Targeting*	8	
*General Sequence Databases*	6	PIR^63^, TCDB^64^, UniProt^65^
**Metabolic and Signaling Pathways**	**58**	
*Protein-Protein Interactions*	30	STITCH^66^, STRING^67^
*Enzymes and Enzyme Nomenclature*	14	CAZy^68^
*Metabolic Pathways*	10	BioCyc^69^, HMDB^70^, MODOMICS^71^, Reactome^72^
*Signaling Pathways*	4	
**Genomics Databases (Non-Vertebrate)**	**47**	
*Prokaryotic Genome Databases*	11	EcoCyc^73^
*Viral Genome Databases*	9	
*Unicellular Eukaryotes Genome Databases*	8	
*Invertebrate Genome Databases*	7	VectorBase^74^
*Fungal Genome Databases*	6	CGD^75^, SGD^76^
*Genome Annotation Terms, Ontologies, and Nomenclature*	4	Genenames^77^
*General Genomics Databases*	2	KEGG^78^, MetaCyc^69^
**Human Genes and Diseases**	**23**	
*General Polymorphism Databases*	5	
*General Human Genetics Databases*	3	
*Gene-, System- or Disease-Specific Databases*	7	
*Cancer Gene Databases*	7	CanSAR^79^
*Human Genes and Diseases*	1	COSMIC^80^
**Other Molecular Biology Databases**	**25**	
*Drugs and Drug Design*	21	ChEMBL^81^
*No Subcategory*	3	
*Molecular Probes and Primers*	1	
**Nucleotide Sequence Databases**	**20**	
*Transcriptional Regulator Sites and Transcription Factors*	11	JASPAR^82^
*Gene Structure, Introns and Exons, Splice Sites*	5	
*International Nucleotide Sequence Database Collaboration*	3	DDBJ^83^, ENA^84^
*Coding and Non-Coding DNA*	1	
**Immunological Databases**	**12**	IEDB^85^, IMGT^86^
**RNA Sequence Databases**	**12**	NONCODE^87^, Rfam^88^
**Human and Other Vertebrate Genomes**	**11**	
*Human ORFs*	5	FANTOM^89^, neXtProt^90^
*Model Organisms, Comparative Genomics*	4	Ensembl^91^, Mouse Genome Database^92^, Rat Genome Database^93^
*Human Genome Databases, Maps and Viewers*	2	UCSC Genome Browser^94^
**Plant Databases**	**7**	
*General Plant Databases*	5	
Arabidopsis thaliana	1	
*Other Plants*	1	
**Proteomics Resources**	**8**	dbPTM^62^
**Microarray Data and** **Other Gene Expression Databases**	**2**	ArrayExpress^95^
**Organelle Databases**	**2**	
wwPDB partners RCSB PDB, PDBe, and PDBj are included in the subcategory *Protein Structure*.		
